# The effectiveness of a new approach using movies in the training of medical students

**DOI:** 10.1007/s40037-015-0208-6

**Published:** 2015-09-07

**Authors:** Patrizia Zeppegno, Carla Gramaglia, Alessandro Feggi, Ada Lombardi, Eugenio Torre

**Affiliations:** Dipartimento Medicina Traslazionale, Institute of Psychiatry, Università del Piemonte Orientale, Corso Mazzini 18, 28100 Novara, Italy

**Keywords:** Cinemeducation, Movies, Medical students

## Abstract

**Introduction:**

The use of movies in medical (particularly psychiatric) education has been often limited to portraits of mental illness and psychiatrists. The Psychiatric Institute of the Università del Piemonte Orientale has a longstanding tradition of working with/on movies according to a method developed by Eugenio Torre, using dynamic images as educational incitements. Our aim is to describe the preliminary results on the impact of this intervention in medical students.

**Methods:**

The cinemeducation project lasted 6 months, and included 12 meetings. Forty randomly selected participants were assessed with: Attitudes Towards Psychiatry Scale (ATP-30), Social Distance Scale (SDS), Interpersonal Reactivity Index (IRI), and Toronto Alexithymia Scale (TAS), both at baseline and after 6 months, when the workshop was concluded.

**Results:**

A significant increase was found in the ATP-30 score, and a reduction of the SDS and IRI-Personal Distress scale scores.

**Conclusions:**

Informal feedback from participants was strongly positive. Preliminary results from the assessment of participants are encouraging. Students’ attitudes towards psychiatry and ability to tolerate anxiety when experiencing others’ distress improved, while stigma decreased. The evocative power of movie dynamic images, developed in the group and integrated with the help of the group leader, can enrich students’ knowledge, both from a cognitive and emotional standpoint.

## Essentials

- The flaws of education offered by medical schools as regards empathic and relational skills are currently being debated, as well as the importance of medical humanities as an adjunct to the traditional educational approach.
- Medical humanities can offer insight into human illness, and broadly speaking into the human condition and suffering, understanding of oneself, and responsibility.
- The cinemeducation intervention proved effective in improving students’ attitudes towards psychiatry, reducing stigma, and improving students’ ability to tolerate their own anxiety when experiencing others’ distress.
- This kind of experience, developed in the group and integrated with the help of the group leader, can turn into knowledge, both from a cognitive and emotional standpoint.

## Introduction

The use of movies in medical (particularly psychiatric) education has often been limited to portraits of mental illness and psychiatrists, despite recent proposals about a broader use for them, for instance to address topics which may be overshadowed by the more technical aspects of education.

The Psychiatric Institute of the Università del Piemonte Orientale, in Novara, Italy has a longstanding tradition of working with/on movies according to a method using dynamic images as educational incitements. In the attempt to find an approach to share and teach emotional experiences, Eugenio Torre developed a method which is rooted in the link between education and the arts, and integrates theoretical and technical issues together with the experience of working in a group [1]. The method is also based on Jung’s and Hillman’s considerations about the connection between images and archetypes, the archetypal experience and learning (both from a cognitive and an emotional standpoint). Dynamic images from movies are discussed and elaborated on in a group context, with the group leader supporting the integration of the emerging contents. Since its development this method has been constantly used in the education and training of medical students, psychiatrists, psychotherapists and, broadly speaking, of people involved in helping professions [2].

The aim of this short report is to describe the preliminary results about the impact of this intervention in a sample of medical students.

## Methods

### The cinemeducation seminars

As described elsewhere in detail [2], selection of movies for the seminars does not require that they are focused on mentally ill characters, but rather that they allow a reflection on helping relationships and on the approach to sufferance.

The current cinemeducation project involved both psychiatry residents and medical students, lasted 6 months, and included 12 meetings to reflect on the doctor-patient relationship and the importance of prejudice in psychiatry. Meetings took place approximately every fortnight, from 7 p.m. to 10–11 p.m. The movies were discussed from a psychological perspective in a group setting allowing brainstorming and sharing processes.

The research project was approved by the Institutional Review Board of the Università del Piemonte Orientale as a part of the research duties of the Counselling Service of the University.

### Assessment

Data were collected anonymously via self-report questionnaires from 40 randomly selected participants. Assessment scales were administered both before (baseline, T0) and after the end of the workshop (T1, after 6 months since baseline).

#### Attitudes Towards Psychiatry-Scale (ATP-30) [3]

The validated 30-item, Likert-type scale is designed to measure medical students’ attitudes to psychiatry. Higher scores indicate a more positive attitude.

#### Social Distance Scale (SDS) [4]

The SDS comprises seven questions that refer to interaction with the target individual, each rated by the subject on a 4-point Likert scale. Higher scores indicate a tendency to maintain a greater social distance from people diagnosed with a mental illness.

#### Interpersonal Reactivity Index (IRI) [5]

Multidimensional scale composed of 28 self-report items, which assess four dimensions of dispositional empathy: Perspective Taking, Fantasy, Empathic Concern, and Personal Distress. Perspective Taking assesses the tendency to spontaneously adopt the psychological point of view of others; Fantasy measures the tendency to imaginatively transpose oneself into the feelings and actions of fictitious characters in books, movies, and plays. The Empathic Concern subscale measures other-oriented feelings of sympathy and concern for others in distress. The Personal Distress subscale assesses self-oriented anxiety when experiencing others in distress.

#### Toronto Alexithymia Scale (TAS) [6]

The TAS-20 consists of three subscales. Factor 1 (F1) assesses the difficulty of identifying feelings; Factor 2 (F2) assesses the difficulty of describing feelings; Factor 3 (F3) assesses externally oriented thinking. The total score ranges from 20 to 100 points, and scores of 61 and above suggest an alexithymic state.

#### Statistical analysis

Data analysis was performed with the Statistical Package for Social Sciences version 20. The T test was used to compare the questionnaires mean scores at T0 and T1.

## Results

Forty students (20 females and 20 males) were randomly selected among participants. Questionnaire scores are reported in Table 1, as well as the T test results. A significant increase was found in the ATP-30 score, and a reduction of the SDS and IRI-PD scores.

**Table 1:** Questionnaire scores and T test *p* values

Questionnaires		Mean	Standard deviation	*p*
ATP-30 T0		95.09	3.477	**0.034**
ATP-30 T1		106.82	15.059	
IRI	F T0	22.73	3.438	**0.163**
	F T1	20.00	5.621	
	EC T0	19.55	1.864	**0.429**
	EC T1	18.45	5.047	
	PT T0	20.82	3.601	**0.438**
	PT T1	19.64	6.345	
	PD T0	19.82	2.483	**0.022**
	PD T1	15.27	5.833	
SDS T0		9.73	3.003	**0.010**
SDS T1		8.00	2.683	
TAS	F1 T0	16.18	5.546	**0.355**
	F1 T1	14.18	6.178	
	F2 T0	16.27	3.495	**0.180**
	F2 T1	14.91	3.618	
	F3 T0	24.82	2.676	**0.387**
	F3 T1	25.73	3.101	

*ATP-30* Attitudes Towards Psychiatry-Scale, *IRI* Interpersonal Reactivity Index, *F* Fantasy, *EC* Empathic Concern, *PT* Perspective Taking, *PD* Personal Distress, *SDS* Social Distance Scale, *TAS* Toronto Alexithymia Scale, *F1, F2, F3* Factor 1–3.

## Conclusions

An educational approach addressing both scientific issues and the humanities (literature, visual arts, films and music) may help medical students to reflect on empathy-related issues, to develop a compassionate approach to patients and their sufferance, and to enhance their awareness about stigmatizing attitudes. This is certainly important in psychiatry education, but should be at the basis of every medical student’s training as well.

Informal feedback from participants to the cinemeducation seminars was strongly positive. The results from the random assessment of participants, albeit preliminary, are encouraging. The intervention proved effective in improving students’ attitudes towards psychiatry, reducing stigma, and strengthening students’ ability to tolerate their own anxiety when experiencing others’ distress. All clinicians will meet psychiatric patients in their clinical practice and are bound to confront themselves with people’s distress and sufferance (both physical and psychological), and with the challenges of helping relationships. From this point of view, an approach using movies may be a useful adjunct to theoretical and technical medical teachings.

Owing to their immediate evocative power, movie dynamic images arouse emotional involvement and this experience, developed in the group and integrated with the help of the group leader, can turn into knowledge, both from a cognitive and emotional standpoint. All these issues are particularly meaningful for doctors-to-be and should not be neglected in medical education.

## References

[1] Torre E. La formazione psicologica degli operatori [Psychological training of professionals]. Paper presented at: The 1st National Congress SIMB; April 2000; Turin.

[2] Gramaglia C, Jona A, Imperatori F, Torre E, Zeppegno P (2013). Cinema in the training of psychiatry residents: focus on helping relationships. BMC Med Educ.

[3] Burra P, Kalin R, Leichner P (1982). The ATP 30-a scale for measuring medical students’ attitudes to psychiatry. Med Educ.

[4] Davis M (1980). A multidimensional approach to individual differences in empathy. JSAS Cat Sel Doc Psychol.

[5] Penn DL, Guynan K, Dally T, Spaulding DW, Garbin CP, Sullivan M (1994). Dispelling the stigma of schizophrenia: what sort of information is best?. Schizophr Bull.

[6] Bagby RM, Parker JDA, Taylor GJ (1994). The twenty-item Toronto Alexithymia Scale—item selection and cross-validation of the factor structure. J Psychosom Res.

